# The Number of X Chromosomes Causes Sex Differences in Adiposity in Mice

**DOI:** 10.1371/journal.pgen.1002709

**Published:** 2012-05-10

**Authors:** Xuqi Chen, Rebecca McClusky, Jenny Chen, Simon W. Beaven, Peter Tontonoz, Arthur P. Arnold, Karen Reue

**Affiliations:** 1 Department of Integrative Biology and Physiology and Laboratory of Neuroendocrinology or the Brain Research Institute, University of California Los Angeles, Los Angeles, California, United States of America; 2 Department of Human Genetics, David Geffen School of Medicine, University of California Los Angeles, Los Angeles, California, United States of America; 3 Department of Medicine, Division of Digestive Diseases, David Geffen School of Medicine, University of California Los Angeles, Los Angeles, California, United States of America; 4 Howard Hughes Medical Institute, David Geffen School of Medicine, University of California Los Angeles, Los Angeles, California, United States of America; 5 Department of Pathology and Laboratory Medicine, David Geffen School of Medicine, University of California Los Angeles, Los Angeles, California, United States of America; 6 Molecular Biology Institute, David Geffen School of Medicine, University of California Los Angeles, Los Angeles, California, United States of America; University of Wisconsin-Madison, United States of America

## Abstract

Sexual dimorphism in body weight, fat distribution, and metabolic disease has been attributed largely to differential effects of male and female gonadal hormones. Here, we report that the number of X chromosomes within cells also contributes to these sex differences. We employed a unique mouse model, known as the “four core genotypes,” to distinguish between effects of gonadal sex (testes or ovaries) and sex chromosomes (XX or XY). With this model, we produced gonadal male and female mice carrying XX or XY sex chromosome complements. Mice were gonadectomized to remove the acute effects of gonadal hormones and to uncover effects of sex chromosome complement on obesity. Mice with XX sex chromosomes (relative to XY), regardless of their type of gonad, had up to 2-fold increased adiposity and greater food intake during daylight hours, when mice are normally inactive. Mice with two X chromosomes also had accelerated weight gain on a high fat diet and developed fatty liver and elevated lipid and insulin levels. Further genetic studies with mice carrying XO and XXY chromosome complements revealed that the differences between XX and XY mice are attributable to dosage of the X chromosome, rather than effects of the Y chromosome. A subset of genes that escape X chromosome inactivation exhibited higher expression levels in adipose tissue and liver of XX compared to XY mice, and may contribute to the sex differences in obesity. Overall, our study is the first to identify sex chromosome complement, a factor distinguishing all male and female cells, as a cause of sex differences in obesity and metabolism.

## Author Summary

Differences exist between men and women in the development of obesity and related metabolic diseases such as type 2 diabetes and cardiovascular disease. Previous studies have focused on the sex-biasing role of hormones produced by male and female gonads, but these cannot account fully for the sex differences in metabolism. We discovered that removal of the gonads uncovers an important genetic determinant of sex differences in obesity—the presence of XX or XY sex chromosomes. We used a novel mouse model to tease apart the effects of male and female gonads from the effects of XX or XY chromosomes. Mice with XX sex chromosomes (relative to XY), regardless of their type of gonad, had increased body fat and ate more food during the sleep period. Mice with two X chromosomes also had accelerated weight gain, fatty liver, and hyperinsulinemia on a high fat diet. The higher expression levels of a subset of genes on the X chromosome that escape inactivation may influence adiposity and metabolic disease. The effect of X chromosome genes is present throughout life, but may become particularly significant with increases in longevity and extension of the period spent with reduced gonadal hormone levels.

## Introduction

Obesity represents a risk factor for many types of metabolic disease, including diabetes, coronary heart disease, osteoarthritis, and even cancer. The study of rare mutations in humans and induced mutations in mouse models has identified numerous genetic factors that influence energy balance [1], [2], [3]. Less is known, however, about common genetic factors that may contribute to moderate differences in body fat storage among individuals in a population [4]. In humans and many other mammals, differences exist between males and females in the amounts and anatomical distribution of fat storage [5], [6], [7], [8], [9]. In general, males tend to have more visceral fat while females have more lower body and subcutaneous fat [10]. The two sexes also differ in the levels of adipose tissue-derived hormones leptin and adiponectin [11], [12], [13], and in the response of fat store depletion to caloric restriction [14]. These differences may contribute to differences between men and women in susceptibility to metabolic disease.

The genetic origins of sex differences in fat tissue accumulation are not well understood. Most studies have focused on the role of gonadal hormones (reviewed in [15], [16]. It is well established that the reduction in levels of estrogens, progestins, and androgens occurring at menopause in women increases central fat accumulation and risk for diabetes, cardiovascular diseases and other disorders [17]. Further evidence that estrogens play an important role in fat metabolism comes from mouse studies. For example, both male and female mice lacking estrogen receptor α have increased white adipose tissue mass and insulin resistance [18]. In men, the accumulation of excess abdominal adipose tissue is associated with low levels of gonadal androgens [19]. Hyperandrogenism is also associated with increased abdominal obesity in women with polycystic ovarian syndrome [20]. Androgen receptor-deficient male mice develop late onset obesity, particularly affecting visceral fat [21], [22]. In addition, the administration of dihydrotestosterone suppresses the development of subcutaneous adipose tissue in wild-type but not androgen receptor-deficient mice [22]. Thus, gonadally derived hormones from both males and females influence body fat, albeit in distinct ways.

Although gonadal hormones are a key determinant of sexual dimorphism in body fat and metabolism, it is notable that even prior to the differentiation of the gonads, human and mouse male embryos are larger than female embryos, suggesting that non-gonadal factors also contribute [23], [24]. In addition to gonadal hormones, an additional fundamental genetic difference exists within every cell in the body of females compared to males (reviewed in [25], [26], [27], [28]. This is the presence in female cells of two X chromosomes, and in male cells of an X and a Y chromosome. The Y chromosome, and specifically the *Sry* gene located there, initiates differentiation of the testes. Mice that have a Y chromosome from which *Sry* has been deleted develop ovaries rather than testes. Conversely, an *Sry* transgene inserted onto autosome is sufficient to convert XX female mice to gonadal males [29]. Inactivation of one X chromosome in each non-germline XX cell greatly reduces the sex difference in level of expression of X genes that is predicted based on the number of copies of X genes [30]. However, a finite set of genes on both mouse and human X chromosomes escape inactivation, and would therefore be expected to exhibit higher expression levels in XX compared to XY cells [31], [32], [33], [34]. Recent studies indicate that genes escaping X chromosome inactivation exhibit elevated expression in metabolic tissues from XX compared to XO mice [35], and could potentially contribute to sex differences in metabolic phenotypes.

In the present study, we utilize the Four Core Genotypes (FCG) mouse model to distinguish between the effects of gonadal sex (testes or ovaries) and sex chromosomes (XX or XY) on adiposity and related metabolic traits [25], [26], [27], [36]. The FCG model allows the generation of gonadal male and female mice carrying either XX or XY sex chromosome complements. Specifically, the FCG Y chromosome sustained a mutation deleting the *Sry* gene (yielding the “Y minus” chromosome, Y^−^), which is complemented in some mice by an *Sry* transgene located on an autosome. Mice having both the Y^−^ chromosome and the *Sry* transgene will develop normally as fertile gonadal male mice. If these mice are bred to a normal female (XX), four types of progeny are produced: female mice with ovaries and XY or XX sex chromosomes (XYF and XXF, respectively), and male mice with testes and XY or XX sex chromosomes (XYM and XXM, respectively). If differences in a trait of interest occur between the gonadal male mice (XYM and XXM) and gonadal female mice (XYF and XXF), it is most likely related to differences in gonadal hormones, although the groups also potentially differ because of possible effects of *Sry* on non-gonadal tissues. By contrast, differences between XX and XY mice suggest a sex chromosome effect, likely directly caused by the difference in number of X or Y chromosomes.

In our study, FCG mice were gonadectomized as adults to remove the acute sex differences resulting from gonadal hormones, and thereby uncover the contribution of sex chromosome complement. We found that gonadectomized XX mice of both gonadal sexes have two-fold increased adiposity compared to XY mice of either gonadal sex. Further genetic studies with mice carrying XO and XXY chromosome complements revealed that the difference is attributable to dosage of the X chromosome, rather than effects of the Y chromosome. These results demonstrate a fundamental difference in adiposity and metabolism conferred by genes on the sex chromosomes, and specifically implicate X chromosome genes as the direct cause of these differences. These results further suggest that X chromosome genes whose expression levels are influenced by dosage or parental imprinting are candidates for metabolic disease differences in men and women.

## Results

### Sex chromosome complement influences body weight and fat mass independent of sex hormones

**Figure 1:**
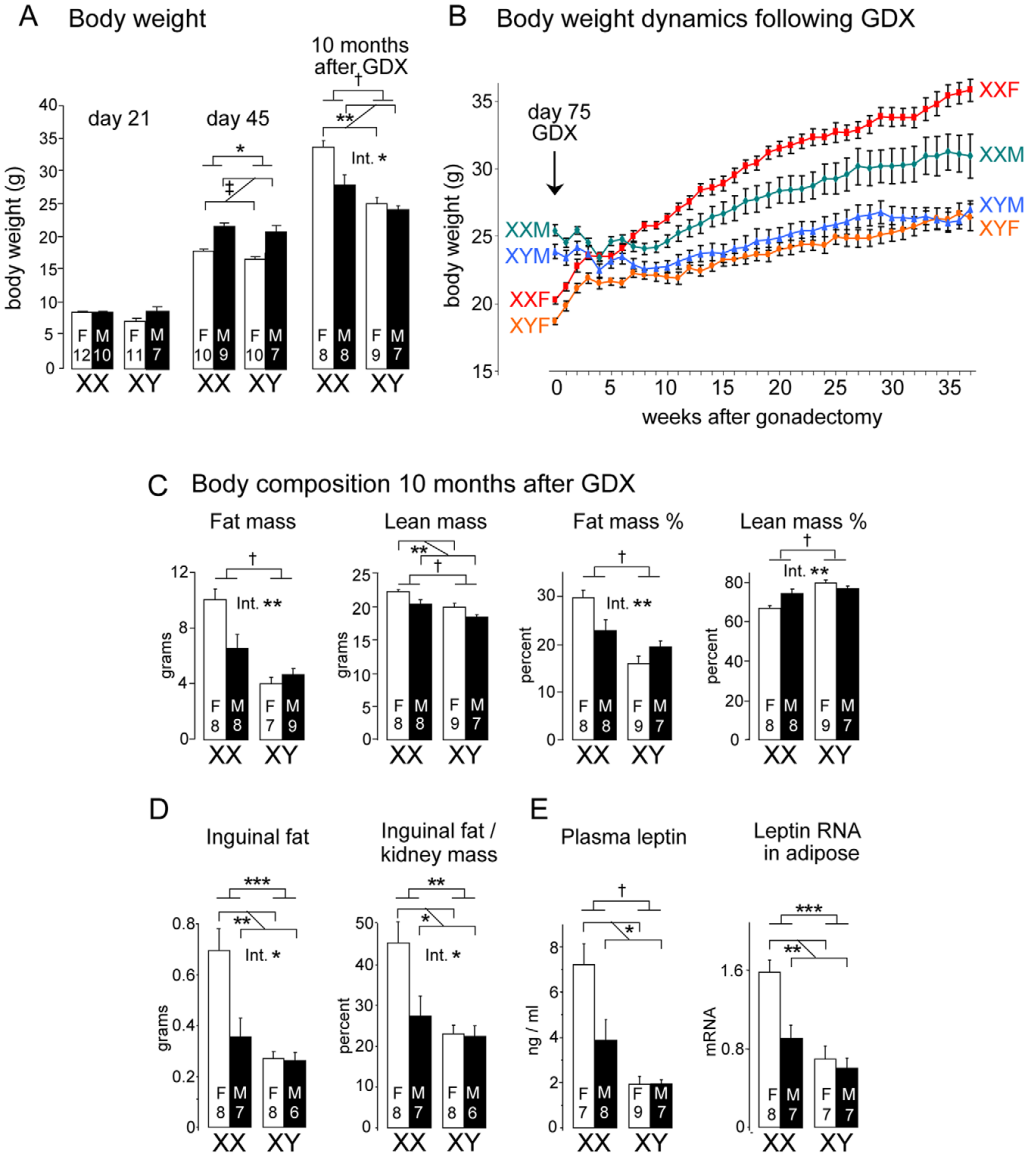
Increased body weight and fat mass in XX versus XY mice on a chow diet. (A) Body weight of four core genotype (FCG) mice at day 21 and day 45, prior to gonadectomy (GDX), and at 10 months after GDX. F, gonadal female; M, gonadal male. Values represent the mean ± SEM for the number of animals indicated in each bar. Significant comparisons for sex chromosome complement and for gonadal sex are denoted by brackets. A significant interaction of sex chromosome complement and gonadal sex is denoted by “Int.”. The p values are as described at the end of the legend. (B) Body weight curve for FCG mice from the point of gonadectomy through 10 months following gonadectomy. Values represent mean ± SEM. Values are significantly different between mice with XX vs. XY genotypes beginning at week 7 following GDX and beyond that. (C) Body composition of mice in panel (B) determined by NMR in FCG mice at 10 months after GDX. (D) Fat pad mass in mice from panel (B) at 10 months following GDX expressed as absolute mass (grams) or relative to kidney weight, which is invariant among the genotypes. (E) Plasma leptin levels and leptin mRNA levels in inguinal adipose tissue. *, p<0.05; **, p<0.01; ***, p<0.001; †, p<0.0001; ‡, p<0.000001.

To determine whether sex chromosome effects contribute to sex differences in body weight and fat mass in adulthood, we examined these traits in C57BL/6 FCG mice (XX gonadal females, XX gonadal males, XY gonadal females, and XY gonadal males). Mice were maintained on a standard chow diet with low fat content (5% by weight). At the time of weaning at postnatal day 21, the four groups of FCG mice did not differ in body weight (Figure 1A). By 45 days of age, gonadal males of either sex chromosome complement were approximately 20% heavier than gonadal females. At 75 days of age the gonadal males were 25% (XX background) or 28% (XY background) heavier than corresponding gonadal females (Figure 1B, time 0). Importantly, however, in these gonadally intact mice there was also a significant influence of sex chromosomes on body weight. At 75 days of age, XX mice were heavier than XY mice by 6.3% (XX>XY gonadal males) and 8.8% (XX>XY gonadal females) (p<0.0001) (Figure 1B, time 0).

Differences observed between male and female gonadally intact FCG mice can be attributed to either activational effects of gonadal hormones (reversible effects caused by sex differences in on-going action of gonadal hormones) or organizational effects (long-lasting or permanent gonadal hormone effects exerted at an earlier stage of development). To distinguish between these alternatives, mice were gonadectomized at 75 days of age to remove activational effects of gonadal hormones. At the time of gonadectomy, male XY and XX mice had significantly higher body weight than female XX and XY mice, although XX mice of either gonadal type weighed more than XY mice, as described above (Figure 1B). In the 4 weeks following gonadectomy (GDX), the body weights of all genotypes converged, and differences between mice that were originally gonadal males and females disappeared (Figure 1B). By 7 weeks, there emerged significant differences based on sex chromosome complement, with XX mice weighing more than XY mice (p<0.000005; Figure 1). At 10 months after GDX, the XX mice weighed 24% more than XY mice (p<0.0001, Figure 1A). In addition, XX gonadal females continued to weigh more than XX gonadal males despite the absence of gonadal secretions for 10 months (Figure 1A, p<0.01 for XX female vs. XX male mice), suggesting an interaction between XX sex chromosome complement and long-acting (organizational) gonadal hormone effects (interaction p<0.05). Thus, the male-female difference in number of X chromosomes influences body weight in the opposite direction to the male-female difference in gonadal hormones.

The increased body weight in XX compared to XY mice reflects a near doubling of the absolute fat mass as ascertained by NMR analysis of whole mice, with 88% higher fat mass in XX compared to XY mice (Figure 1C). When expressed as a percent of body weight, XX mice had 50% higher proportional fat mass than XY mice (p<0.00001, Figure 1C). This dramatic difference in fat mass between XX and XY mice is particularly striking considering that the mice were fed a standard mouse chow diet with very low fat content. XX mice also had slightly higher lean body mass than XY mice (Figure 1C). The increased total body adiposity of XX compared to XY mice was reflected in isolated fat pad mass (Figure 1D; p<0.0005 for absolute fat pad mass, p<0.005 for mass relative to kidney; kidney weight did not differ among genotypes). Fat mass, percent lean mass, and fat pad mass all exhibited significant sex chromosome effects, and also significant interactions between sex chromosome and gonadal sex (indicated in Figure 1A, 1C and 1D by ‘Int’).

In parallel with the increased adiposity, plasma leptin levels were elevated 2–3-fold in XX compared to XY mice (p<0.00005) (Figure 1E). Plasma leptin was also higher in females (p<0.05), but only in XX mice (interaction p<0.05). This suggests that long lasting gonadal effects, as well as genetic factors conferred by sex chromosome complement, directly or indirectly influenced leptin levels. Leptin mRNA levels in adipose tissue mirrored plasma leptin levels, with highest levels in XX mice (p<0.0005 vs. XY mice), and significantly higher levels in mice that previously had ovaries rather than testes (p<0.01) (Figure 1E).

### XX mice exhibit increased daytime food intake preceding increased body weight

To identify metabolic differences that could contribute to the increased adiposity of XX mice, we measured food intake, physical activity, and energy expenditure parameters while mice were housed in metabolic cages. We performed these studies at two ages: (1) at 4 weeks following gonadectomy, at which time the body weights for all four genotypes were similar and measurements were not complicated by differences in body weight or composition, and (2) at 10 months after gonadectomy, after body weight differences in XX vs. XY mice were pronounced (see Figure 1B).

**Figure 2:**
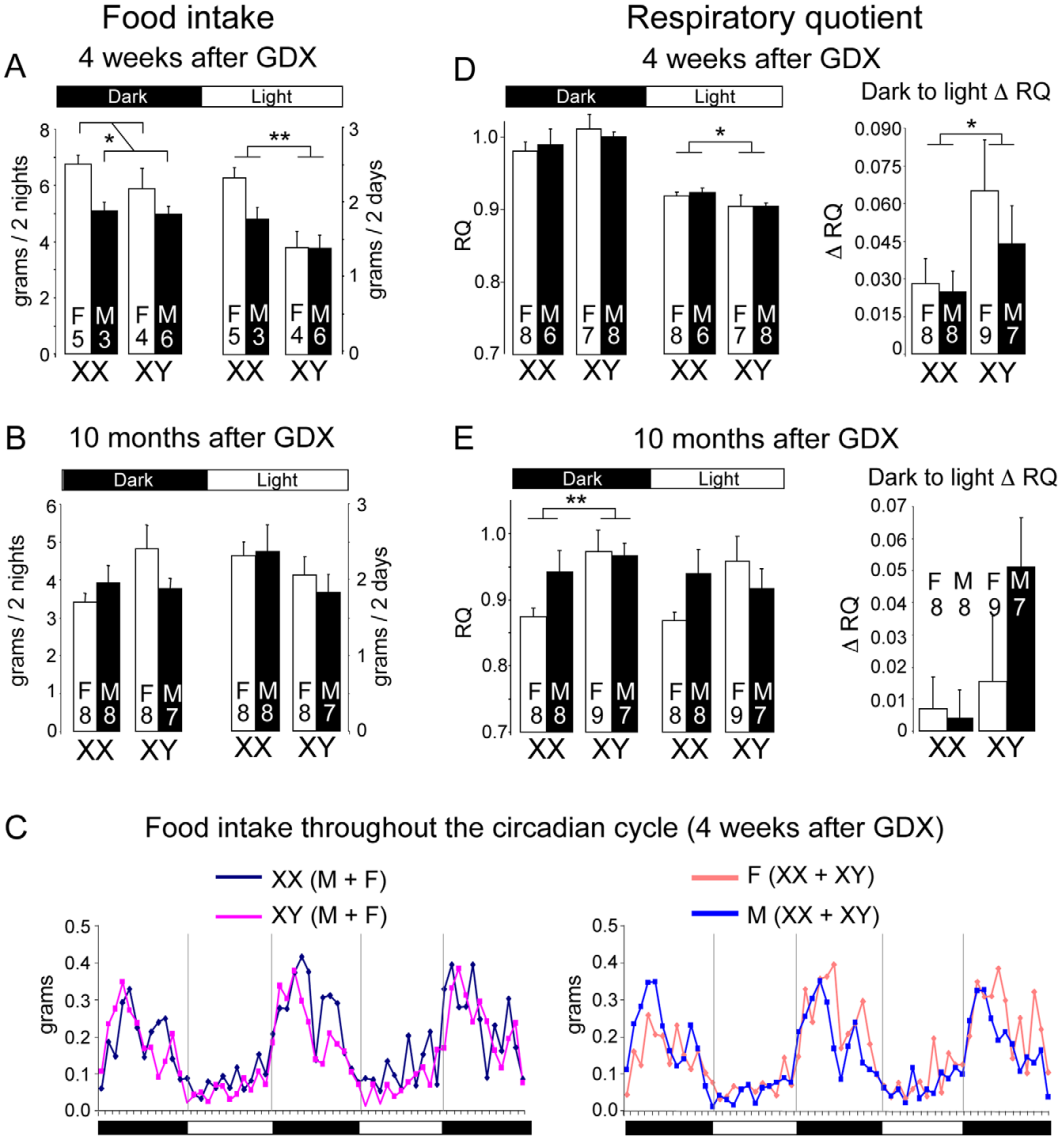
Altered food intake and RQ in XX versus XY mice. Mice were individually housed in metabolic cages to assess food take and energy balance parameters continually throughout the diurnal cycle. (A, B) Food intake determined at 4 weeks or 10 months following GDX. Values represent the mean ± SEM food intake summed over two dark or two light periods. Data shown represent raw values; normalization to body weight or to lean body mass gave the same outcome. (C) Food intake patterns throughout 3 nights and 2 days. At left, lines represent mean values for XX (n = 8) and XY (n = 10) mice; at right, lines represent mean values for gonadal females (n = 9) and gonadal males (n = 9). (D, E) Mean Respiratory Quotient (RQ) ± SEM for light and dark periods determined at 4 weeks or 10 months following GDX. At right, the change in RQ between dark and light periods (Δ RQ) is shown. *, p<0.05; **, p<0.01; ***, p<0.001; ‡, p<0.000001.

At 4 weeks post-GDX, we detected a difference among the genotypes in food intake patterns monitored continuously throughout the circadian cycle. During the dark period when mice typically consume 70% of total calories [37], gonadal female mice of both XX and XY chromosome complements consumed more than gonadal males (*p*<0.05; Figure 2A). Since these measurements were made only 4 weeks after GDX, this may reflect lingering effects of gonadal secretions. However, during the daytime, food intake was significantly higher in XX females and males compared to XY mice (*p*<0.01; Figure 2A and 2C). Since this difference occurred at an age when no differences exist in body weight, the increased daytime food intake is likely to contribute to subsequent divergence of body weight between XX and XY mice. At 10 months after GDX, the average absolute food intake for all genotypes was reduced compared to values at 4 weeks post-GDX, but no significant differences in food intake were observed among the four genotypes (Figure 2B).

Using indirect calorimetry, we detected significant sex chromosome effects on respiratory quotient (RQ), a measure of the relative reliance on carbohydrate (RQ = 1) and fat substrates (RQ = 0.7) as metabolic fuel. At four weeks post-GDX, all mice exhibited the expected diurnal variation in RQ, with highest values in the dark phase. Notably, however, XX mice maintained a significantly higher RQ than XY mice during the light phase (Figure 2D), which may be related to the differential feeding pattern in XX mice (Figure 2A). In addition, compared to XY mice, XX mice exhibited a smaller amplitude change in RQ from dark to light periods (Δ RQ), suggesting reduced flexibility in fuel switching (Figure 2D). By contrast, at 10 months post-GDX, after XX mice had accumulated nearly twice as much adipose tissue as XY mice, the pattern of fuel utilization had changed. At this point, the XX mice had lower RQ than XY mice during the dark phase (Figure 2E), indicating increased fat utilization in the fed state, possibly an adaptive change in response to the excess fat storage.

Besides food intake and RQ, other energy balance parameters did not differ significantly among the four genotypes. These include oxygen consumption (which was assessed per mouse, per lean body mass [38], and via linear regression [39] to account for contributions of both lean and fat mass in energy metabolism), thermogenic gene expression, and physical activity in the horizontal and vertical planes (Figure S1). Thus, the key differences in energy metabolism between XX and XY mice were increased daytime food intake and reduced flexibility in RQ in XX mice. Both of these were apparent prior to the divergence in body weight.

Despite the greater adiposity, XX mice did not exhibit substantially impaired glucose homeostasis. At four weeks after GDX, fasting glucose levels were higher in gonadal female than gonadal male mice (p<0.0001), and slightly higher in XX than XY mice (p<0.05); there were no differences in fasting insulin levels among the genotypes (Figure S2A). At ten months after GDX when XX mice had considerably greater adiposity, glucose and insulin levels were similar among the four genotypes, and no differences were revealed by glucose tolerance test (Figure S2A). The ability to maintain glucose homeostasis despite excess fat storage in the XX mice may be related to adaptive changes in metabolism in these mice. For example, at ten months after GDX when XX mice had substantially higher fat mass, they exhibited increased expression of fatty acid oxidation genes encoding acyl CoA oxidase (*Aox1*) and carnitine palmitoyl transferase (*Cpt1*) in both muscle and liver (Figure S2B). Increased fatty acid oxidation may reduce the extent of lipid accumulation in liver and skeletal muscle, and prevent impaired glucose homeostasis.

### Enhanced weight gain, dyslipidemia, and fatty liver in XX mice on a high fat diet

**Figure 3:**
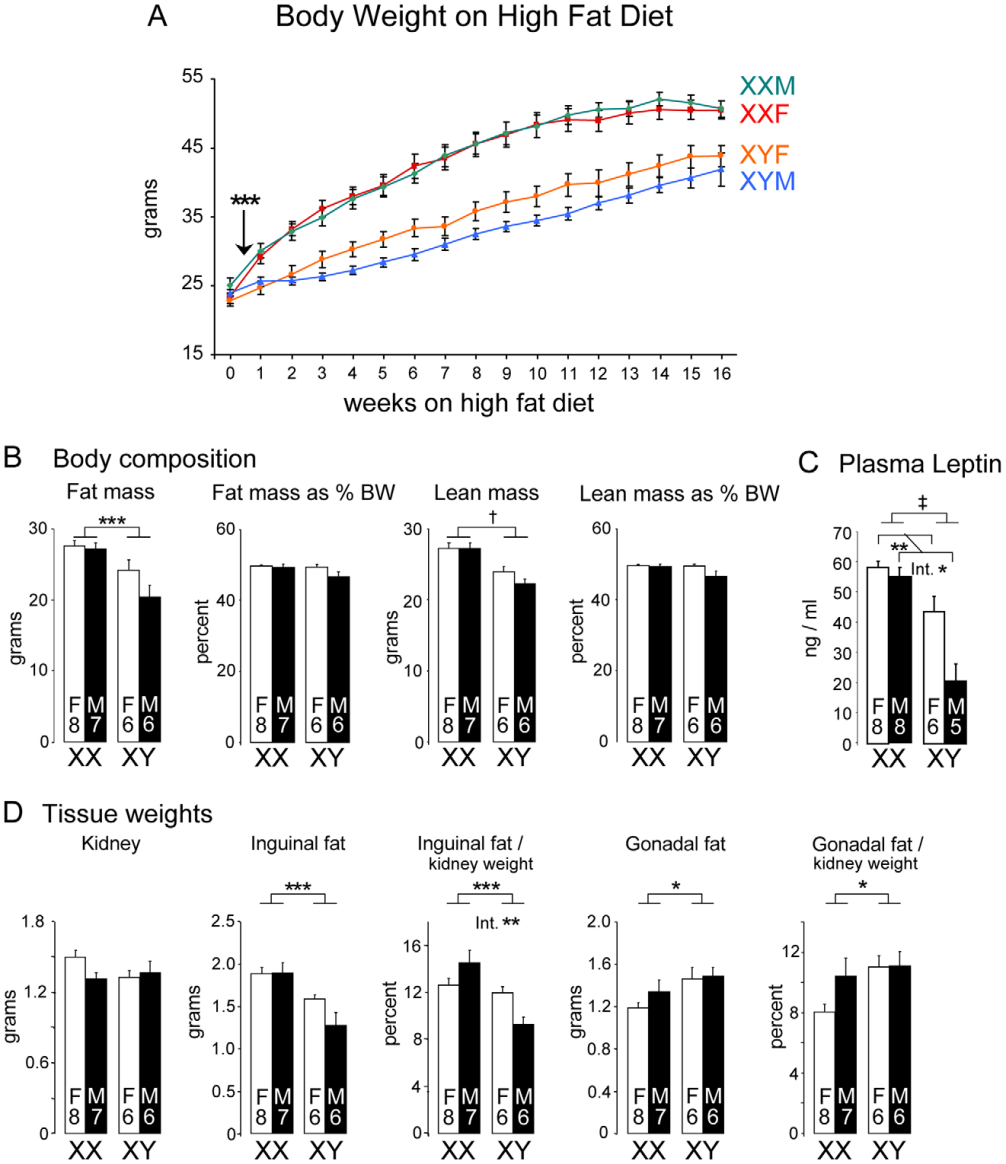
Enhanced weight gain and fat mass in XX compared to XY mice fed a high fat-high carbohydrate diet. (A) FCG mice were fed a high fat-high carbohydrate diet beginning at 4 weeks following GDX (week 0). XX mice first exhibited significantly higher body weight beginning at 3 days on the diet (arrow), and throughout the remainder of the study. (B) Body composition was determined by NMR at 16 weeks of the high fat-high carbohydrate diet. Fat and lean mass are shown as absolute mass and as percent of total body weight (BW). (C) Plasma leptin levels were higher in XX mice than XY mice at the end of the 16 week high fat diet period, and higher in gonadal females than gonadal males. (D) Tissue weights were determined by excision after 16 weeks on the high fat-high carbohydrate diet. Kidney weight did not differ among the four genotypes. Inguinal and gonadal fat pads are shown as absolute weights and normalized to kidney weight. Values shown for all bars represent mean ± SEM for the number of each genotype indicated. *, p<0.05; **, p<0.01; ***, p<0.001; †, p<0.0001; ‡, p<0.000001.

As described above, on a chow diet containing minimal fat, XX mice accumulate excess adipose tissue without impaired glucose homeostasis. Metabolic dysregulation in human obesity typically occurs in the presence of a more stressful nutritional environment. We hypothesized that a combination of sex chromosome complement and a high fat diet may make XX mice more vulnerable to metabolic dysregulation than XY mice. To test this, we placed FCG on a high fat, simple carbohydrate diet that promotes weight gain [40]. Mice were gonadectomized at 75 days of age, continued on a chow diet for 4 weeks, and then fed the high fat diet for 16 weeks. As shown in Figure 3A, the mice of all four genotypes had similar body weight at the beginning of the high fat diet. However, within just 3 days of beginning the high fat diet, the XX and XY mice diverged, with significantly higher body weight in XX gonadal males and females than in the corresponding XY mice (p<0.005). XX mice continued to gain weight at an accelerated pace throughout most of the 16 weeks, and weighed about 15% more than XY mice at the end of the diet (p<0.000005). The enhanced weight gain on the high fat diet appeared to obscure the male-female difference in XX mice that was observed on the chow diet (Figure 1B).

NMR assessment of body composition showed that after 16 weeks on the high fat diet, XX mice had higher absolute fat mass than XY mice (p<0.005), but the increase in fat mass was not significant when expressed as a proportion of body weight (Figure 3B). Nevertheless, the increased fat mass was reflected in elevated plasma leptin levels in XX compared to XY mice (p<0.000001); leptin levels were also significantly higher in female vs. male mice (p<0.01; interaction of sex by sex chromosome complement p<0.05) (Figure 3C). Absolute lean mass was also increased in XX mice (p<0.00005), but not when expressed as a proportion of body weight (Figure 3B). Thus, it appears that the greater increase in body weight observed in XX compared to XY mice on a high fat diet is attributable to increases in both fat and lean mass, and that XX mice exhibit increased absolute fat mass and circulating leptin levels.

The analysis of tissue weights of mice after 16 weeks on the high fat diet revealed sex chromosome effects on the liver and adipose tissue depots. Absolute kidney weight did not differ among the four genotypes despite differences in body weight (Figure 3D), and was used to normalize the weights of other tissues. Inguinal subcutaneous fat pads weighed more in XX compared to XY mice (Figure 3D; absolute weight, p<0.0005; normalized to kidney, p<0.001). We also detected a sex chromosome by gonadal sex interaction in inguinal fat pad weight when normalized to kidney weight (p = 0.006), suggesting a role for organizational hormone action in combination with XX or XY status in determining subcutaneous fat pad expansion on a high fat diet. Unexpectedly, the gonadal fat depot showed the opposite pattern, with slightly higher values in XY mice expressed both as absolute weight (p<0.05) and normalized weight (p<0.05) (Figure 3D). These results indicate that distinct genetic and hormonal factors may influence the expansion of the gonadal and inguinal fat depots on a high fat diet.

**Figure 4:**
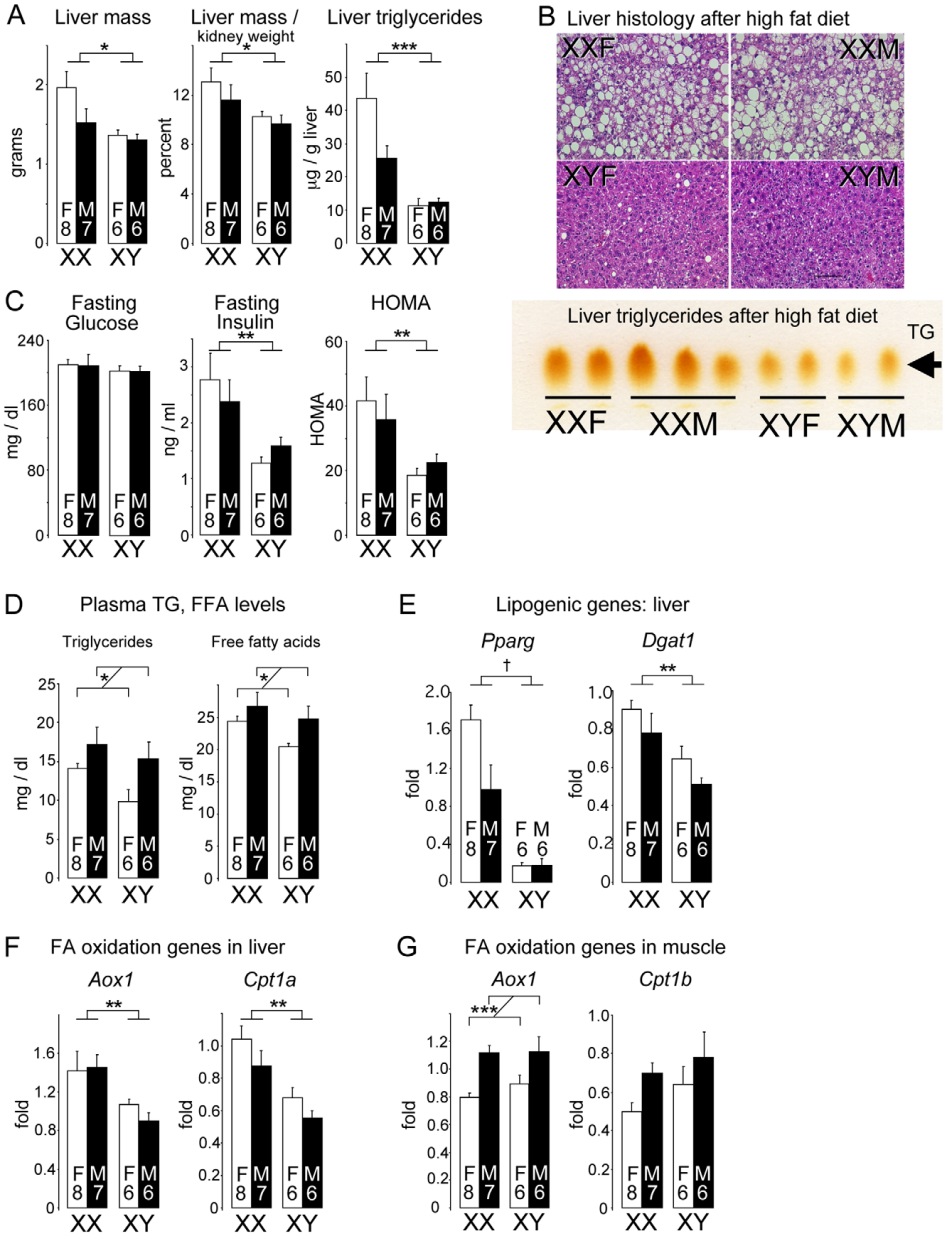
Diet-induced fatty liver and impaired glucose homeostasis are more pronounced in XX than XY mice. FCG mice were fed a high fat-high carbohydrate diet for 16 weeks. All values represent mean ± SEM. (A) XX mice had increased liver mass and hepatic triglyceride levels compared to XY mice. (B) Hematoxylin and eosin staining of liver sections shows hepatic lipid droplet accumulation in XX mice. In the lower panel, thin layer chromatography demonstrates increased triglyceride levels in liver of XX mice relative to XY mice. (C) Fasting glucose and insulin levels, and homeostatic model assessment (HOMA) were determined. XX mice had significantly higher insulin levels than XY mice. (D) Fasting plasma triglyceride (TG) and free fatty acid (FFA) levels were higher in gonadal males than in gonadal females. (E) Increased triglyceride accumulation in XX liver is associated with increased peroxisome proliferator-activated receptor γ (*Pparg*) and diacylglycerol acyltransferase 1 (*Dgat1*) mRNA levels. (F) Increased expression levels for fatty acid oxidation genes acyl CoA oxidase 1 (*Aox*1) and carnitine palmitoyltransferase 1α (*Cpt1a*) in liver of XX mice. (G) Increased expression of Aox1 in skeletal muscle of gonadal male mice. *, p<0.05; **, p<0.01; ***, p<0.001; †, p<0.0001.

The high fat diet elicited formation of a fatty liver specifically in XX mice. XX mice exhibited a significant increase in liver weight, an abundance of lipid droplets, and increased triglyceride content (p<0.0005, XX vs. XY mice) (Figure 4A, 4B). The XX mice also exhibited evidence of reduced insulin sensitivity, as fasting insulin levels and HOMA were elevated 2-fold compared to XY mice in the presence of similar glucose levels (Figure 4C). These metabolic disturbances were not associated with increased circulating triglyceride or free fatty acid levels in XX mice, which instead differed between gonadal males and females (Figure 4D). This suggests that triglyceride and fatty acid levels are influenced by organizational hormone effects rather than sex chromosome complement, and are not likely an underlying factor in the development of the fatty liver in XX vs. XY mice.

Gene expression in liver of mice fed the high fat diet showed enhanced expression of lipogenic factors, including the transcription factor peroxisome proliferator-activated receptor γ, and the triglyceride biosynthetic enzyme diacylglycerol acyltransferase 1 (Figure 4E). Hepatic expression of genes encoding proteins involved in fatty acid uptake (Cd36), fatty acid synthesis (fatty acid synthase), and fatty acid desaturation (stearoyl CoA desaturase) were not significantly different among the four genotypes (data not shown). Despite the increased triglyceride accumulation, fatty acid oxidation gene expression was also elevated in XX compared to XY liver (Figure 4F; p<0.005). This pattern was also observed in XX mice fed the chow diet (Figure S2B), and may represent an adaptive or compensatory response that prevents even more pronounced fat storage in XX mice. In contrast to liver, *Aox1* and *Cpt1b* mRNA levels in muscle correlated with gonadal sex rather than sex chromosome complement (Figure 4G). Metabolic gene expression is clearly under complex control, with sex chromosomes and gonadal sex effects having differing roles in specific tissues and conditions. Overall, our results reveal that the XX chromosome complement led to accelerated weight gain and less desirable metabolic profile than XY mice in response to a high fat diet.

### The number of X chromosomes, not the presence of the Y chromosome, determines differences in body weight and adiposity

XX mice differ genetically from XY mice in both the dose of the X chromosome and in the absence of a Y chromosome. We analyzed body weight and fat mass in mouse strains with abnormal Y chromosomes that allow the dissection of effects of X and Y chromosome number. As described below, our results indicate that the XX vs. XY difference is caused by genes on the X chromosome and not the Y chromosome.

**Figure 5:**
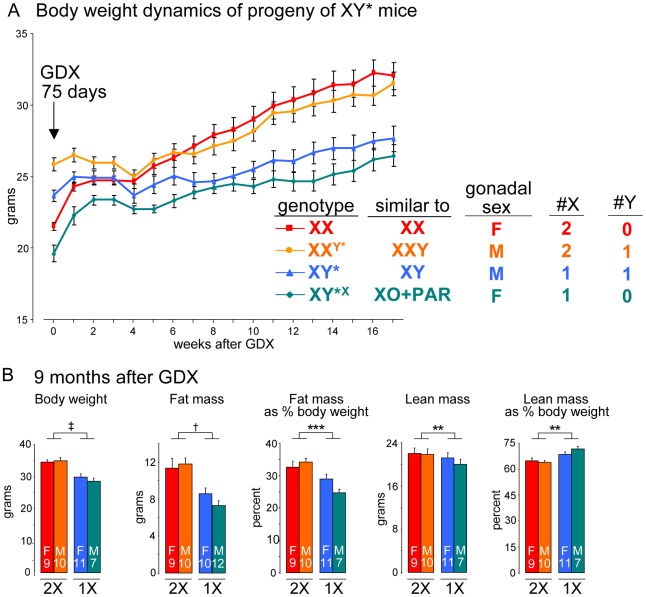
The number of X chromosomes determines differences in body weight and adiposity. (A) Body weight following gonadectomy at day 75 of mice having the indicated sex chromosome complements fed a chow diet. The Y* chromosome has been described [41], and the near equivalent genotype in terms of X and Y chromosome complement is shown. Two groups of mice with two X chromosomes had higher body weight, relative to mice with one X chromosome, beginning at 8 weeks after GDX and thereafter (p<0.000001). Mice with a Y chromosome did not differ from mice lacking a Y chromosome. (B) Body weight and body composition determined by NMR in mice shown in panel (A) at 9 months after GDX. Values shown for all bars represent mean ± SEM for the number of each genotype indicated. **, p<0.01; ***, p<0.001; †, p<0.0001; ‡, p<0.000001.

We took advantage of mice carrying an unusual Y chromosome, Y*, that undergoes abnormal recombination with the X chromosome, producing mice with aberrant numbers of X or Y chromosomes [25], [41]. Thus, by breeding XY* fathers, we obtain progeny with the following genotypes: XX, XX^Y*^ (similar to XXY), XY* (similar to XY), and XY*^X^ (similar to XO+an extra pseudoautosomal region, PAR) (see Table S1). After gonadectomy at day 75, mice with two X chromosomes (XX and XXY) had higher body weight (p<0.000001) and fat mass (p<0.0005) than mice with one X chromosome (XY and XO+PAR) (Figure 5A, 5B). The presence of the Y chromosome appeared to have no effect. We conclude that the inherent genetic difference conferred by presence of two X chromosomes is responsible for the effects on body weight and adiposity.

### Elevated expression levels of genes escaping X chromosome inactivation in adipose tissue and liver

**Figure 6:**
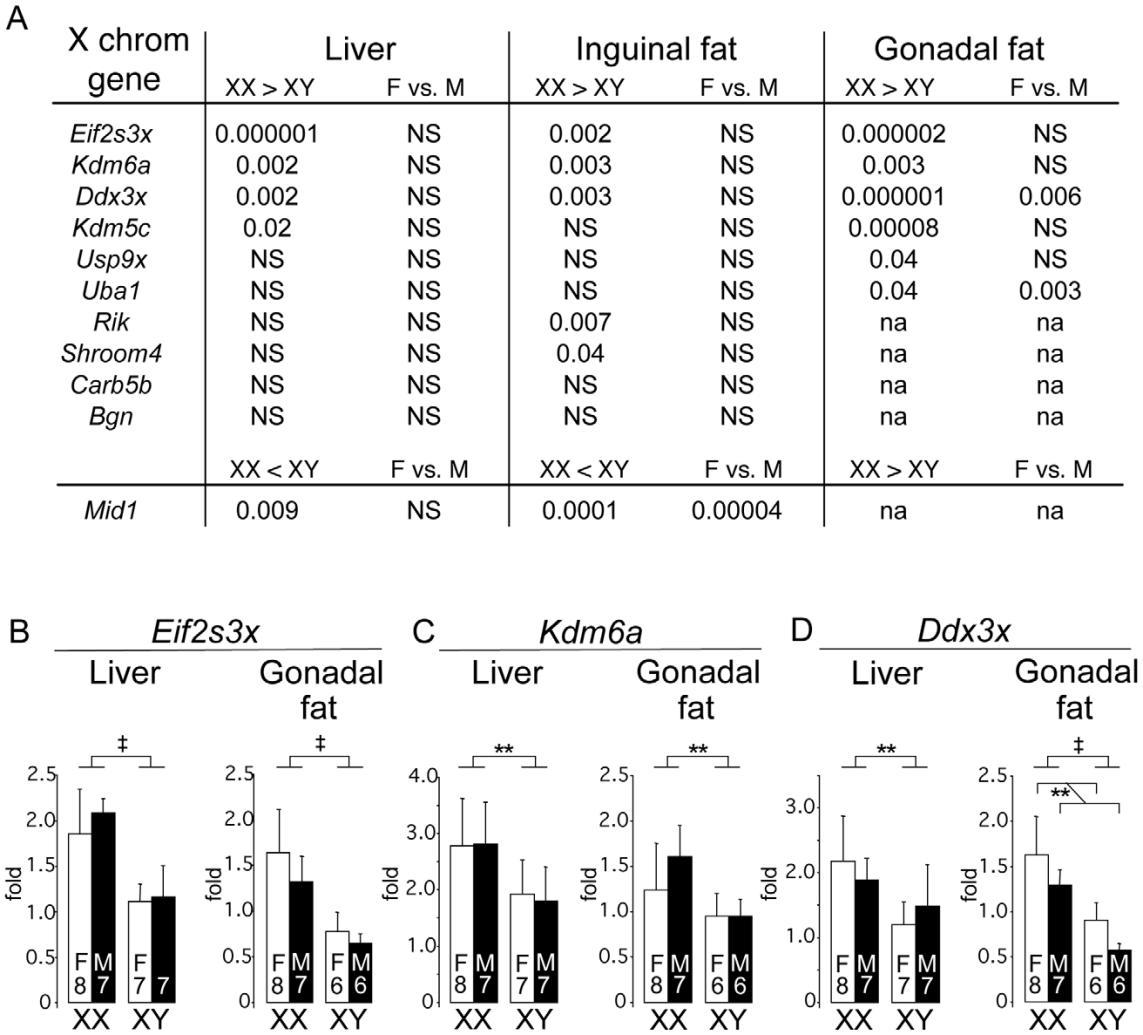
Differential gene expression in liver and fat tissues of X chromosome genes that escape inactivation. Genes previously shown to escape X chromosome inactivation (see text) were assessed for expression levels by quantitative PCR in liver and adipose tissue (subcutaneous inguinal and gonadal depots) of gonadectomized, chow fed FCG mice shown in Figure 1B (10 months post-GDX). (A) Statistical differences in gene expression levels among the FCG genotypes for genes escaping X chromosome inactivation. For each gene, the p value for differences between XX and XY, and female (F) vs. male (M), are shown. Several escapees exhibit increased expression in liver and/or adipose tissues of XX compared to XY mice; a few genes also exhibit differences between levels in gonadal females and gonadal males. *Mid1* shows a unique pattern, with lower expression levels in XX compared to XY tissues. The full name for *Rik* is 2610029G23Rik. NS, not significantly different. (B–D) mRNA levels are shown for liver and gonadal fat tissue of FCG mice for *Eif2s3x*, *Kdm6a*, and *Ddx3x*. Values shown for all bars represent mean ± SEM for the number of each genotype indicated. **, p<0.01; ‡, p<0.000001.

A potential mechanism underlying the observed effect of two X chromosomes on adiposity is the presence of a higher dose of X chromosome genes in XX compared to XY cells. Although X inactivation prevents most X genes from being expressed at higher levels in females, it is well established that a proportion of X chromosome genes in both mouse and human escape inactivation [31], [32], [33], [34]. If genes that escape X chromosome inactivation are expressed at higher levels in metabolic tissues of XX than XY mice, they may contribute to the differences that we have observed between XX and XY mice. We evaluated the expression levels in adipose tissue depots and liver of the FCG mice for protein-coding genes that have been shown to escape X inactivation in an interspecific female mouse cell line, or are candidate “escapees” from X-inactivation because of higher expression in XX vs. XO mice, or XX vs. XY mice (listed in Figure 6A) [34], [35], [42], [43].

We found that 8 of 11 genes in our panel exhibited higher expression levels in XX compared to XY mouse adipose tissues (gonadal and/or inguinal) (Figure 6A). These include four genes that are established X escapees in both mouse and human (*Eif2s3x*, *Kdm6a*, *Ddx3x*, *Kdm5c*), and these genes also show higher expression in gonadectomized XX liver as well as adipose tissue (Figure 6A–6D). Another gene that is also known to escape inactivation in mouse and human, *Mid1*, exhibited a unique expression pattern, with significantly lower expression in XX compared to XY inguinal fat and liver. The mechanism for this reduced expression in XX tissues is unclear, but nevertheless constitutes a difference that is determined by sex chromosome complement. Only a handful of genes (*Ddx3x*, *Uba1*, *Mid1*) showed significant differences in expression levels between gonadal female and male mice, which may reflect long-lasting effects of gonadal hormones on expression levels of these genes. These results reveal that a subset of genes escaping X inactivation are expressed at elevated levels in metabolic tissues of XX compared to XY mice. These genes represent candidates for future studies to identify the mechanism by which increased X chromosome dosage affects adiposity and metabolism.

## Discussion

Sexual dimorphism occurs in many fundamental metabolic processes, which likely influence the development of metabolic diseases. Understanding the sex-specific factors and pathways that promote or mitigate disease may lead to a better understanding of disease pathogenesis and useful interventions. The present results illustrate the complex interplay between several major classes of sex-specific factors that cause sexual dimorphism in obesity, and highlight the utility of the FCG model for investigating such interactions. For the first time, we demonstrate that sex chromosome complement, independent of gonadal sex, has substantial effects on body weight and adiposity in adult mice on a chow diet, and on the rate of weight gain in mice fed a high fat diet. We found that the increased adiposity observed in XX mice is attributable to the presence of two X chromosomes rather than to the lack of a Y chromosome. These results focus attention of future studies on a specific set of X chromosome genes that exhibit altered expression in metabolic tissues of XX compared to XY animals because of escape from X chromosome inactivation or sex chromosome-specific imprinting.

The role of sex hormones in the determination of body weight and adiposity has been documented in many studies in humans and rodent models. For example, gonadally intact male mice have higher body weight, and exhibit more pronounced diet-induced weight gain, than females; this sex difference is reversed partially or completely by ovariectomy of female mice [44]. In humans, the loss of estrogens with menopause is associated with deposition of visceral body fat, and this effect can be ameliorated to some extent by hormone replacement therapy [45], [46], . Modulating testosterone levels also affects adipose tissue storage in healthy young men, with testosterone levels inversely correlated with adipose tissue mass [49]. Thus, it is clear that gonadal hormones play a strong role in determining sex differences in adiposity in mice and humans. However, few models have allowed the interrogation of potential genetic effects underlying sex differences independent of gonadal hormones.

In our characterization of the FCG mice, body weight and/or metabolic traits were influenced by all three of the major classes of sex-biasing factors: activational (acute) hormonal effects, long-lasting (organizational) hormonal effects, and sex chromosome effects [25], [28]. Several traits were influenced by interactions between two or more of these factors. At 75 days of age, gonadal males weighed 25–28% more than gonadal females, irrespective of their sex chromosome complement, suggesting that the sex difference is caused by gonadal secretions. That conclusion was confirmed because the sex difference disappeared by one month after gonadectomy. However, further analysis of the FCG model revealed that attributing sex differences in body weight solely to gonadal hormones would be a significant oversimplification. Prior to gonadectomy, XX mice weighed 6–9% more than XY mice, in both gonadal males and females. The XX vs. XY difference became dramatically larger after gonadectomy, with XX mice having up to 2-fold greater adiposity than XY mice. Layered on top of this was an effect of *Sry* (likely mediated by long-lasting effects of the original gonadal hormones), as without their gonads, gonadal female XX mice lacking *Sry* had higher body weight, fat pad mass, and plasma leptin levels than gonadal male XX mice possessing *Sry*. The results indicate that although sex chromosome effects act in both intact and gonadectomized mice, gonadal hormones blunt the influence of sex chromosome complement, suggesting that the hormones may have different effects depending on the chromosomal sex of cells. Thus, understanding how males and females differ from one another is not simply a matter of studying an apparently dominant factor that causes the sex difference, but requires disentangling the interactive effects of several sex-biasing factors.

The increased body weight of XX mice was preceded by increased food intake compared to XY mice; interestingly, this was restricted to the light portion of the diurnal cycle (see Figure 2A). After differences in adiposity were established between XX and XY mice, food intake was not distinguishable, but leptin levels were elevated in XX mice, suggesting relative leptin resistance in the XX mice. Since there were no detectable compensatory changes in energy expenditure or physical activity in XX mice, this increase in food intake likely contributes to the increased body weight. The increased consumption of carbohydrates during the light period was reflected in slightly elevated RQ during the same period. This difference was evident even before the GDX XX mice had increased body weight. A trend toward increased food intake during the light period continued after the XX mice were substantially heavier (at 10 months post-GDX), although it was no longer statistically significant. A recent study has shown that mouse food intake during the light period of the circadian cycle leads to greater weight gain than equivalent intake during the dark period, when mice typically consume the majority of their calories [50]. Many other studies have provided evidence that energy balance is tightly integrated with the circadian clock and that disruption of this cycle has detrimental effects on many aspects of metabolism [51], [52]. Thus, a focus of future studies in the FCG model will be the investigation of whether sex chromosome complement influences regulation of the circadian clock and/or networks for food consumption and satiety.

Sex chromosome complement had a key effect on the response to a high fat diet, with XX mice having an almost immediate divergence in weight gain from XY mice. An interesting finding was the greater expansion in the subcutaneous fat depot in XX mice, and greater increase in the gonadal fat depot in XY mice. It has been shown that women store a greater percentage of dietary fatty acids in subcutaneous adipose tissue than men [53]. Our observations in mice raise the possibility that sex chromosome complement may be a factor in determining the propensity to store fat in various anatomical depots.

The high fat diet also led to the development of more pronounced metabolic dysregulation in XX mice, particularly fatty liver. Non-alcoholic fatty liver disease affects up to one-third of American adults, usually in association with obesity and insulin resistance [54], [55]. The occurrence of fatty liver disease and its progression to cirrhosis, end-stage liver disease and hepatocellular carcinoma are influenced by many factors, including genetic factors. Our studies reveal that XX sex chromosome complement is one genetic factor that promotes development of fatty liver in mice. It is likely that the fatty liver in XX mice fed the high fat diet was influenced by risk factors such as increased adiposity and hyperinsulinemia. Interestingly, however, fatty liver did not parallel circulating triglyceride and fatty acid levels, which were more influenced by gonadal sex (likely influenced by organizational effects of gonadal hormones) rather than sex chromosome complement. In future studies, it will be interesting to determine whether sex chromosome complement also influences the propensity for progression of steatotic livers to cirrhosis, the basis of which is currently not understood.

The sex chromosome effects reported here indicate that inherent sex differences in expression of X chromosome genes, stemming from the difference in number or parental imprint of X genes in XX vs. XY mice, contribute to sex differences in adiposity and metabolic disease. The sex chromosome effects are unlike typical linkage of genes to phenotype, because they are not caused by differences in the genetic sequence of the X chromosome, which was identical in all mice studied. Because X-inactivation effectively reduces the inherent bias toward higher expression of X genes in XX mice relative to XY mice, prime candidates for the genes causing the adiposity are those that escape X inactivation, or those that receive a parental imprint, leading to differential expression in XX compared to XY mice [28].

A significant proportion of X chromosome genes (15–25%) are thought to escape X chromosome inactivation in humans [56], and most of the genes escaping X inactivation in mice also escape in humans [34]. We tested expression levels of candidate genes that are known to escape inactivation in both mouse and human (*Eif2s3x*, *Kdm6a*, *Kdm5c*, *Ddx3x*) or have a Y paralogue with some evidence for higher expression in XX than XY mice and humans (*Usp9x*, *Uba1*) [31], [34], [35], [42], [43], [57]. Each of these genes was expressed at higher levels in XX than XY gonadal fat in gonadectomized mice, providing evidence that these genes escape inactivation in a metabolic tissue. Thus, these genes are candidates for those causing the XX–XY differences in physiology and adiposity reported here. Alternatively, differential expression of X chromosomes escapee genes could occur secondarily to differences in adiposity between XX and XY mice, in which case they may be downstream players in the observed metabolic differences. In addition to sex chromosome genes, autosomal genes that are differentially expressed in response to X chromosome gene dosage may contribute to the observed metabolic differences between XX and XY mice. It is known, for example, that the dysregulation of genes involved in mitochondrial metabolism and protein translation occurs in tissues of XX compared to XO mice, but the metabolic consequences are not known [35].

A reasonable question is whether these studies in the mouse have relevance to obesity in humans. Unusual numbers of sex chromosomes in human conditions such as Klinefelter (XXY) and Turner (XO) syndromes are associated with metabolic disease and/or adiposity [58], [59], [60], [61]. However, in these diseases, endocrine abnormalities may contribute and are difficult to distinguish from the sex chromosome effects. The utility of our model is that it is genetically tractable in a way that human studies are not. Since fundamental genetic and metabolic processes are shared between mice and humans, we propose that the identification of X-linked genes that have a large impact on obesity in the mouse could lead to the discovery of novel mechanisms that impact obesity in humans. The increasing longevity of the human population means that the hypogonadal period may extend for up to half of a persons' lifetime, and the inherent genetic sex differences uncovered here may have important ramifications. Furthermore, since the gene content of the X chromosome is conserved in mouse and human, and several of the same genes escape inactivation in both species, there is hope that characterizing the action of X gene(s) in mouse will advance our understanding of human metabolic disease.

## Materials and Methods

### Ethics statement

All animals were handled in strict accordance with good animal practice as defined by the relevant national and/or local animal welfare bodies, and all animal work was approved by the appropriate committee. All experiments in this paper were carried out with UCLA IACUC approval.

### Mice

“Four core genotypes” (FCG) mice were used. “Male” denotes a mouse with testes, and “female” denotes a mouse with ovaries. In these mice, the testis-determining *Sry* gene is deleted from the Y chromosome and inserted as a transgene onto an autosome [27], [36]. Thus, gonadal type is no longer controlled by sex chromosome complement (XX vs. XY), and the effect of sex chromosome complement on traits can be studied independent of the gonadal type of the mouse. For the present study, the FCG model was transferred to a C57BL/6J (B6) genetic background by backcrossing male MF1 XY^−^ *Sry* (Y^−^ chromosome denotes deletion of *Sry*; *Sry* denotes presence of the autosomal *Sry* transgene) to B6 XX females for 13–14 generations. Four groups of mice are generated, XX and XY gonadal males (XXM and XYM, carrying the *Sry* transgene), and XX and XY gonadal females (XXF and XYF, without *Sry*). In all the FCG mice the Y^−^ chromosome derives from strain 129. Advantages and caveats in the use of FCG mice have been discussed [25], [27].

Gonadectomy was performed at 75 days of age. Under isoflurane anesthesia, mice were given a subcutaneous injection of carprofen and the gonads were removed. Using aseptic procedures, gonads were exposed, clamped, ligated, and excised. Successful gonadectomy was confirmed at the time of euthanasia. Although no gonadal hormones are present in GDX mice, sex steroid hormones (*e.g.*, androgens or estrogens produced *de novo* in adrenal, adipose tissue, or brain) are probably present in mice after GDX.

In one study, we compared mice born of XY* fathers, which have an aberrant Y chromosome that recombines abnormally with the X chromosome. The XY* males from strain B6Ei.LT-Y*/EiJ from the Jackson Laboratories were crossed with B6/J females for 2–3 generations, so that the mice were a mixture of C57BL/6J and C57BL/EiJ strains. In all case littermates were compared, so that the percentage of the two B6 parental strains was comparable across groups. We studied four different types of progeny of XY*: XX, XX^Y*^, XY*, and XY*^X^. These mice are roughly similar to XX, XXY, XY, and XO+an extra pseudoautosomal region, respectively (see Table S1) [41].

Gonadal males and females were housed in separate cages and maintained at 23°C with a 12∶12 light∶dark cycle. For studies using chow fed mice, mice were fed Purina 5001 chow diet (approximately 5% fat, PMI Nutrition International, St. Louis, MO) throughout their lifetime. For high fat diet treatment, mice were gonadectomized at 75 days of age and 4 weeks later were switched from chow to a high fat diet containing 35% fat, 33% carbohydrate (Diet F3282, Bio-Serve, Frenchtown, New Jersey). Fresh diet was added to cages twice per week. Animal studies were performed under approval of the UCLA Institutional Animal Care and Use Committee.

### Genotyping and karyotyping

DNA was extracted from tails using Chelex resin (Bio-Rad, Hercules, CA). The genotype of mice was determined by PCR based on the presence or absence of *Sry* and of the X/Y chromosome paralogues Jarid1d/Jarid1c [41]. Ear fibroblasts from offspring of XY* mice were cultured and metaphase spreads were used to determine the sex chromosome status based on karyotype [41].

### Measurement of body weight and body composition

FCG and XY* mice were weighed on postnatal days 21, 45 and day 75 and then gonadectomized (GDX) on day 75. After GDX mice were weighed at weekly intervals. At various ages, body composition was determined with a Mouse Minispec apparatus (Bruker Woodlands, TX) with Echo Medical Systems (Houston, TX) software. This apparatus uses NMR spectroscopy for fat and lean mass measurements with coefficients of variation of <3% [62]. Correlation between NMR and gravimetric measurements is better than 0.99.

### Energy balance measurements

Eight calibrated Oxymax metabolic cages (Columbus Instruments) were used to detect numerous variables related to energy balance: food and water intake, horizontal and vertical physical activity, heat production, oxygen consumption, CO_2_ production, energy expenditure, and respiratory quotient (RQ). The room housing the metabolic cages was kept very quiet to avoid stress or other interference with the activity of the mice. Mice were housed individually in the Oxymax metabolic cages from midday Friday to midday Monday, during which parameters were monitored dynamically at 20 min intervals. Mice had free access to water and food resented from a food hopper attached to a scale. Data for 3 full nights and 2 full days were analyzed.

### Glucose homeostasis

Baseline glucose and insulin levels were determined after a 4.5-hour fast (8:00AM–12:30PM). Glucose tolerance tests were performed after similar fast by injecting mice intraperitoneally with glucose (2 mg/g body weight) and determining glucose levels (using Lifescan OneTouch glucose meter) at 15, 30, 60 and 180 minutes after injection [63].

### Quantitative RT–PCR

Liver, quadriceps skeletal muscle, gonadal fat, inguinal fat and brown fat were dissected out rapidly, flash frozen in liquid nitrogen, and stored at −80°C. RNA was isolated from tissues using Trizol (Invitrogen, Carlsbad, CA, USA) and treated with RNase-free DNase (Promega, Madison, USA) to remove possible genomic DNA contamination. First-strand cDNA synthesis was generated by reverse transcription with SuperScript III RNase H-RT (Invitrogen). Quantitative real time PCR (n = 7–8 per genotype) was performed on an ABI 7300 Sequence Detection system (Applied Biosystems, Foster City, CA, USA) using the SensiMix*Plus*SYBR Green & Fluorescein Master Mix Kit (Quantace, USA). Two or three control genes were amplified as normalization controls: beta-2 microglobulin, TATA box-binding protein (TBP), and BC022960. Primer sequences for all genes assessed are listed in Table S2. Cycling conditions were: 95°C for 10 min; 40 cycles of 95°C for 15 sec, 60°C for 30 sec and 72°C for 30 sec. assay contained a standard curve for the target gene and control genes with 4 serial dilution points of control cDNA: 50 ng, 10 ng, 2 ng and 0.4 ng. Dissociation curves were examined to eliminate the possibility of genomic DNA contamination.

### Statistical analyses

Groups were compared using two-way ANOVA (NCSS 2001; Number Cruncher Statistical Systems, Kaysville, UT, USA) with main factors of sex (gonadal male vs. gonadal female, same as *Sry* present vs. absent) and sex chromosome complement (XX vs. XY). Sometimes a three-way repeated measures ANOVA was also applied with between factors of sex and sex chromosome complement, and within factors of gonadal status (before vs. after GDX) or age. Statistical analyses (main effects of each of the two factors, or interaction of the two) are presented if they were statistically significant, but usually not if they were not significant (p>0.05). Multiple regression analyses of energy metabolism data was performed with Stata Data Analysis and Statistical Software (StataCorp LP, College Station, TX).

## Supporting Information

Figure S1 Energy metabolism measurements in FCG mice fed a chow diet. Oxygen consumption (VO_2_) and physical activity along horizontal and vertical axes were determined in mice individually housed in metabolic cages at 4 weeks (A) and 10 months (B) following GDX. No significant differences were detected among the four genotypes in these parameters during dark or light cycles. (C) Thermogenic gene expression was assessed in brown adipose tissue at 4 weeks and 10 months following GDX. Uncoupling protein 1 (*Ucp1*) gene expression was higher in male than female mice at 4 weeks after GDX, but this difference was no longer apparent at 10 months. No significant differences in peroxisome proliferator-activated receptor γ coactivator α (*Pgc1*) mRNA levels were detected. Each bar represents mean ± SEM for the number of mice of each genotype indicated. **, p<0.01.
(DOC)

Figure S2 Glucose homeostasis and gene expression in FCG mice fed a chow diet. (A) Glucose and insulin levels were determined after a 4 hour fast (08:00–12:00). Glucose tolerance was assessed by intraperitoneal injection of glucose and blood collection at intervals over 3 hours. The GTT AUC represents the area under the curve of blood glucose levels plotted from time 0 to 3 hours post glucose injection. (B) Gene expression determined by qPCR for acyl CoA oxidase 1 (*Aox1*) and carnitine palmitoyltransferase (*Cpt*) in muscle and liver, as indicated. Each bar represents mean ± SEM for the number of mice of each genotype indicated. *, p<0.05; **, p<0.01; †, p<0.0001.
(DOC)

Table S1 Sex chromosome composition of offspring from XY* x XX mice. The copy number of specific regions of the X and Y chromosomes present in mice of each genotype is indicated. NPX, non-pseudoautosomal region of the X chromosome. MSY, male-specific region of the Y chromosome. Xm, maternal X imprint. Xp, paternal X imprint. Refer to [41] for illustrations of chromosome structures.
(DOC)

Table S2 Primer sequences for gene expression analyses by qPCR.
(DOC)

## References

[1] Blakemore AI, Froguel P (2010). Investigation of Mendelian forms of obesity holds out the prospect of personalized medicine.. Ann N Y Acad Sci.

[2] Donkor J, Reue K, Leff T, Granneman J (2010). Mouse models of lipodystrophy.. Adipose Tissue in Health and Disease.

[3] O'Rahilly S (2009). Human genetics illuminates the paths to metabolic disease.. Nature.

[4] Lusis AJ, Attie AD, Reue K (2008). Metabolic syndrome: from epidemiology to systems biology.. Nat Rev Genet.

[5] Kotani K, Tokunaga K, Fujioka S, Kobatake T, Keno Y (1994). Sexual dimorphism of age-related changes in whole-body fat distribution in the obese.. Int J Obes Relat Metab Disord.

[6] Lovejoy JC, Champagne CM, de Jonge L, Xie H, Smith SR (2008). Increased visceral fat and decreased energy expenditure during the menopausal transition.. Int J Obes (Lond).

[7] Macotela Y, Boucher J, Tran TT, Kahn CR (2009). Sex and depot differences in adipocyte insulin sensitivity and glucose metabolism.. Diabetes.

[8] Power ML, Schulkin J (2008). Sex differences in fat storage, fat metabolism, and the health risks from obesity: possible evolutionary origins.. Br J Nutr.

[9] Wajchenberg BL (2000). Subcutaneous and visceral adipose tissue: their relation to the metabolic syndrome.. Endocr Rev.

[10] Nielsen S, Guo Z, Johnson CM, Hensrud DD, Jensen MD (2004). Splanchnic lipolysis in human obesity.. J Clin Invest.

[11] Cnop M, Havel PJ, Utzschneider KM, Carr DB, Sinha MK (2003). Relationship of adiponectin to body fat distribution, insulin sensitivity and plasma lipoproteins: evidence for independent roles of age and sex.. Diabetologia.

[12] Combs TP, Berg AH, Rajala MW, Klebanov S, Iyengar P (2003). Sexual differentiation, pregnancy, calorie restriction, and aging affect the adipocyte-specific secretory protein adiponectin.. Diabetes.

[13] Havel PJ, Kasim-Karakas S, Dubuc GR, Mueller W, Phinney SD (1996). Gender differences in plasma leptin concentrations.. Nat Med.

[14] Shi H, Strader AD, Woods SC, Seeley RJ (2007). Sexually dimorphic responses to fat loss after caloric restriction or surgical lipectomy.. Am J Physiol Endocrinol Metab.

[15] Brown LM, Gent L, Davis K, Clegg DJ (2010). Metabolic impact of sex hormones on obesity.. Brain Res.

[16] Pallottini V, Bulzomi P, Galluzzo P, Martini C, Marino M (2008). Estrogen regulation of adipose tissue functions: involvement of estrogen receptor isoforms.. Infect Disord Drug Targets.

[17] Garaulet M, Perez-Llamas F, Baraza JC, Garcia-Prieto MD, Fardy PS (2002). Body fat distribution in pre-and post-menopausal women: metabolic and anthropometric variables.. J Nutr Health Aging.

[18] Heine PA, Taylor JA, Iwamoto GA, Lubahn DB, Cooke PS (2000). Increased adipose tissue in male and female estrogen receptor-alpha knockout mice.. Proc Natl Acad Sci U S A.

[19] Blouin K, Boivin A, Tchernof A (2008). Androgens and body fat distribution.. J Steroid Biochem Mol Biol.

[20] Dunaif A (1997). Insulin resistance and the polycystic ovary syndrome: mechanism and implications for pathogenesis.. Endocr Rev.

[21] Fan W, Yanase T, Nomura M, Okabe T, Goto K (2005). Androgen receptor null male mice develop late-onset obesity caused by decreased energy expenditure and lipolytic activity but show normal insulin sensitivity with high adiponectin secretion.. Diabetes.

[22] Sato T, Matsumoto T, Yamada T, Watanabe T, Kawano H (2003). Late onset of obesity in male androgen receptor-deficient (AR KO) mice.. Biochem Biophys Res Commun.

[23] Bukowski R, Smith GC, Malone FD, Ball RH, Nyberg DA (2007). Human sexual size dimorphism in early pregnancy.. Am J Epidemiol.

[24] Burgoyne PS, Thornhill AR, Boudrean SK, Darling SM, Bishop CE (1995). The genetic basis of XX-XY differences present before gonadal sex differentiation in the mouse.. Philos Trans R Soc Lond B Biol Sci.

[25] Arnold AP (2009). Mouse models for evaluating sex chromosome effects that cause sex differences in non-gonadal tissues.. J Neuroendocrinol.

[26] Arnold AP, Burgoyne PS (2004). Are XX and XY brain cells intrinsically different?. Trends Endocrinol Metab.

[27] Arnold AP, Chen X (2009). What does the “four core genotypes” mouse model tell us about sex differences in the brain and other tissues?. Front Neuroendocrinol.

[28] Arnold AP (2011). The end of gonad-centric sex determination in mammals.. Trends Genet.

[29] Goodfellow PN, Lovell-Badge R (1993). SRY and sex determination in mammals.. Annu Rev Genet.

[30] Itoh Y, Melamed E, Yang X, Kampf K, Wang S (2007). Dosage compensation is less effective in birds than in mammals.. J Biol.

[31] Berletch JB, Yang F, Disteche CM (2010). Escape from X inactivation in mice and humans.. Genome Biol.

[32] Brown CJ, Greally JM (2003). A stain upon the silence: genes escaping X inactivation.. Trends Genet.

[33] Prothero KE, Stahl JM, Carrel L (2009). Dosage compensation and gene expression on the mammalian X chromosome: one plus one does not always equal two.. Chromosome Res.

[34] Yang F, Babak T, Shendure J, Disteche CM (2010). Global survey of escape from X inactivation by RNA-sequencing in mouse.. Genome Res.

[35] Lopes AM, Burgoyne PS, Ojarikre A, Bauer J, Sargent CA (2010). Transcriptional changes in response to X chromosome dosage in the mouse: implications for X inactivation and the molecular basis of Turner Syndrome.. BMC Genomics.

[36] De Vries GJ, Rissman EF, Simerly RB, Yang LY, Scordalakes EM (2002). A model system for study of sex chromosome effects on sexually dimorphic neural and behavioral traits.. J Neurosci.

[37] Ellacott KL, Morton GJ, Woods SC, Tso P, Schwartz MW (2010). Assessment of feeding behavior in laboratory mice.. Cell Metab.

[38] Butler AA, Kozak LP (2010). A recurring problem with the analysis of energy expenditure in genetic models expressing lean and obese phenotypes.. Diabetes.

[39] Kaiyala KJ, Schwartz MW (2011). Toward a more complete (and less controversial) understanding of energy expenditure and its role in obesity pathogenesis.. Diabetes.

[40] Surwit RS, Kuhn CM, Cochrane C, McCubbin JA, Feinglos MN (1988). Diet-induced type II diabetes in C57BL/6J mice.. Diabetes.

[41] Chen X, Watkins R, Delot E, Reliene R, Schiestl RH (2008). Sex difference in neural tube defects in p53-null mice is caused by differences in the complement of X not Y genes.. Dev Neurobiol.

[42] Xu J, Burgoyne PS, Arnold AP (2002). Sex differences in sex chromosome gene expression in mouse brain.. Hum Mol Genet.

[43] Xu J, Taya S, Kaibuchi K, Arnold AP (2005). Sexually dimorphic expression of Usp9x is related to sex chromosome complement in adult mouse brain.. Eur J Neurosci.

[44] Grove KL, Fried SK, Greenberg AS, Xiao XQ, Clegg DJ (2010). A microarray analysis of sexual dimorphism of adipose tissues in high-fat-diet-induced obese mice.. Int J Obes (Lond).

[45] Gambacciani M, Ciaponi M, Cappagli B, Piaggesi L, De Simone L (1997). Body weight, body fat distribution, and hormonal replacement therapy in early postmenopausal women.. J Clin Endocrinol Metab.

[46] Haarbo J, Marslew U, Gotfredsen A, Christiansen C (1991). Postmenopausal hormone replacement therapy prevents central distribution of body fat after menopause.. Metabolism.

[47] Lee CG, Carr MC, Murdoch SJ, Mitchell E, Woods NF (2009). Adipokines, inflammation, and visceral adiposity across the menopausal transition: a prospective study.. J Clin Endocrinol Metab.

[48] Van Pelt RE, Jankowski CM, Gozansky WS, Schwartz RS, Kohrt WM (2005). Lower-body adiposity and metabolic protection in postmenopausal women.. J Clin Endocrinol Metab.

[49] Woodhouse LJ, Gupta N, Bhasin M, Singh AB, Ross R (2004). Dose-dependent effects of testosterone on regional adipose tissue distribution in healthy young men.. J Clin Endocrinol Metab.

[50] Arble DM, Bass J, Laposky AD, Vitaterna MH, Turek FW (2009). Circadian timing of food intake contributes to weight gain.. Obesity.

[51] Bass J, Takahashi JS (2010). Circadian integration of metabolism and energetics.. Science.

[52] Huang W, Ramsey KM, Marcheva B, Bass J (2011). Circadian rhythms, sleep, and metabolism.. J Clin Invest.

[53] Romanski SA, Nelson RM, Jensen MD (2000). Meal fatty acid uptake in adipose tissue: gender effects in nonobese humans.. Am J Physiol Endocrinol Metab.

[54] Cohen JC, Horton JD, Hobbs HH (2011). Human fatty liver disease: old questions and new insights.. Science.

[55] Kopec KL, Burns D (2011). Nonalcoholic fatty liver disease: a review of the spectrum of disease, diagnosis, and therapy.. Nutr Clin Pract.

[56] Carrel L, Willard HF (2005). X-inactivation profile reveals extensive variability in X-linked gene expression in females.. Nature.

[57] Johnston CM, Lovell FL, Leongamornlert DA, Stranger BE, Dermitzakis ET (2008). Large-scale population study of human cell lines indicates that dosage compensation is virtually complete.. PLoS Genet.

[58] Bakalov VK, Cheng C, Zhou J, Bondy CA (2009). X-chromosome gene dosage and the risk of diabetes in Turner syndrome.. J Clin Endocrinol Metab.

[59] Bardsley MZ, Falkner B, Kowal K, Ross JL (2011). Insulin resistance and metabolic syndrome in prepubertal boys with Klinefelter syndrome.. Acta Paediatr.

[60] Bojesen A, Kristensen K, Birkebaek NH, Fedder J, Mosekilde L (2006). The metabolic syndrome is frequent in Klinefelter's syndrome and is associated with abdominal obesity and hypogonadism.. Diabetes Care.

[61] Van PL, Bakalov VK, Bondy CA (2006). Monosomy for the X-chromosome is associated with an atherogenic lipid profile.. J Clin Endocrinol Metab.

[62] Taicher GZ, Tinsley FC, Reiderman A, Heiman ML (2003). Quantitative magnetic resonance (QMR) method for bone and whole-body composition analysis.. Anal Bioanal Chem.

[63] Vergnes L, Beigneux AP, Davis R, Watkins SM, Young SG (2006). Agpat6 deficiency causes subdermal lipodystrophy and resistance to obesity.. J Lipid Res.

