# Supplementary material for: The Number of X Chromosomes Causes Sex Differences in Adiposity in Mice
Source: PLoS Genet. 2012 May 10;8(5):e1002709. doi: 10.1371/journal.pgen.1002709 (PMC3349739; doi:10.1371/journal.pgen.1002709)
Supplement: Table S1 — Sex chromosome composition of offspring from XY* x XX mice. The copy number of specific regions of the X and Y chromosomes present in mice of each genotype is indicated. NPX, non-pseudoautosomal region of the X chromosome. MSY, male-specific region of the Y chromosome. Xm, maternal X imprint. Xp, paternal X imprint. Refer to [41] for illustrations of chromosome structures. (DOC) [file pgen.1002709.s003.doc]

**SUPPLEMENTAL TABLE 1**

**Offspring from XY* x XX mice.**

NPX, non-pseudoautosomal region of the X chromosome. MSY, male-specific region of the Y chromosome. Xm, maternal X imprint. Xp, paternal X imprint.
