# Supplementary material for: The Number of X Chromosomes Causes Sex Differences in Adiposity in Mice
Source: PLoS Genet. 2012 May 10;8(5):e1002709. doi: 10.1371/journal.pgen.1002709 (PMC3349739; doi:10.1371/journal.pgen.1002709)
Supplement: Table S2 — Primer sequences for gene expression analyses by qPCR. (DOC) [file pgen.1002709.s004.doc]

SUPPLEMENTAL TABLE 2. qPCR primers used for gene expression analysis

| **Genes** | **primers** |  |  |  |  |  |  |  |  |
| --- | --- | --- | --- | --- | --- | --- | --- | --- | --- |
| *β2m* (beta-2 microglobulin) | forward: 5'-TGGTGCTTGTCTCACTGACC-3'  reverse: 5'-GTATGTTCGGCTTCCCATTC-3' | | | | | | | |  |
| *Tbp* (TATA box binding protein ) | forward: 5’-ACCCTTCACCAATGACTCCTATG-3’  reverse: 5’ ATGATGACTGCAGCAAATCGC-3’ | | | | | | | | |
| *Bc022960* (Mus musculus cDNA BC022960 ) | forward: 5’-ATCCCCAAGATCATCAGCAG-3’  reverse: 5’-TTCGTTAGCCCACTTGCTTT-3’ | | | | | | | |  |
| *Aox1* (acyl CoA oxidase 1) | forward: 5’-CAGGAAGAGCAAGGAAGTGG-3’  reverse: 5’-CCTTTCTGGCTGATCCCATA-3’ | | | | | | | |  |
| *Cpt1a (*carnitine palmitoyltransferase Ia) | forward: 5' AAACCCACCAGGCTACAGTG-3'  reverse: 5'-TCCTTGTAATGTGCGAGCTG-3' | | | | | | | |  |
| *Cpt1b (*carnitine palmitoyltransferase Ib) | forward: 5’-GTCGCTTCTTCAAGGTCTGG-3’  reverse: 5’- AAGAAAGCAGCACGTTCGAT-3’ | | | | | | | |  |
| *Dgat1*(diacylglycerol O-acyltransferase 1) | forward 5’-TGCTACGACGAGTTCTTGAG-3’  reverse: 5’-CTCTGCCACAGCATTGAGAC-3’ | | | | | | | |  |
| Leptin | forward 5'-GCTCCAGCAGCTGCAAGGTG-3’  reverse: 5’-AAGTCCAAGCCAGTGACCCTC-3’ | | | | | | | | |
| Adiponectin | forward: 5’-GGAACTTGTGCAGGTTGGAT-3’  reverse: 5’-GCTTCTCCAGGCTCTCCTT-3’ | | | | | | | |  |
| *Ucp1* (uncoupling protein1) | forward: 5’-GGGCCCTTGTAAACAACAAA-3’  reverse: 5’-GTCGGTCCTTCCTTGGTGTA-3’ | | | | | | | |  |
| *Pparγ* (peroxisome proliferator activated receptor gamma) | forward: 5’-CCAGAGCATGGTGCCTTCGCT-3’  reverse: 5’-CAGCAACCATTGGGTCAGCTC-3' | | | | | | | | |
| *Pgc1α* (Pparγ, coactivator 1 alpha) | forward: 5’-CTCACAGAGACACTGGACAGT-3’  reverse: 5’-TGTAGCTGAGCTGAGTGTTGG-3' | | | | | | | | |
| *Cd 36* (CD36 antigen) | forward: 5’-TGCAGCTGTTATTGGTGCAG-3’  reverse: 5’-TGGGTTTTGCACATCAAAG-3' | | | | | | | |  |
| *Fasn (fatty acid synthase)* | forward: 5’-CGTGTTGGCCTACACCCAGAG-3’  reverse: 5’-GGCAGCAGGGCCTCCAGCAC-3' | | | | | | | | |
| *Scd1* (stearoyl-Coenzyme A desaturase 1) | forward: 5’-GGT GATGTTCCAGAGGAGGTA-3’  reverse: 5’-GGTGCTAACGAACAGGCT-3' | | | | | | | |  |
| *Ddx3x* | forward:5'-GGATCACGGGGTGATTCAAGAGG-3'  reverse:5'-CTATCTCCACGGCCACCAATGC-3' | | | | | | | | |
| *Kdm5c* | forward: 5’-ACCCACCTGGCAAAAACATTGG-3’  reverse: 5’-ACTGTCGAAGGGGGATGCTGTG-3' | | | | | | | | |
| *Kdm6a* | forward: 5’-CCAATCCCCGCAGAGCTTACCT-3’  reverse: 5’-TTGCTCGGAGCTGTTCCAAGTG-3' | | | | | | | | |
| *Eif2s3x* | forward: 5’-TTGTGCCGAGCTGACAGAATGG-3’  reverse: 5’-CGACAGGGAGCCTATGTTGACCA-3' | | | | | | | | |
| *Usp9x* | forward: 5’-GCATGTCAGCGATTTTTCCGAGA-3’  reverse: 5’-CACATAGCTCCACCAGGCGATG-3' | | | | | | | | |
| *Uba1* | forward: 5’-ACACTGGGCCTCTTGTCGAGGA-3’  reverse: 5’-CAGGCCTCTTGTATCTGCCACCA-3' | | | | | | | |  |
| *Mid1* | forward: 5’-GCGATCATCAGGTGGCAGCTT-3’  reverse: 5’-TTTGGCTTCTTGACGGGATGC-3’ | | | | | | | |  |
| *Car5b* | forward: 5’-CATTGATGCTTGGGGCTCTGA-3’  reverse: 5’-CAGCCAAGCCATTTTCTTCCA-3’ | | | | | | | |  |
| *Shroom4* | forward: 5’-CAGGAAGGCAGCCATACAGGA-3’  reverse: 5’-TCTCAATGCAAGGTCGGAAGG-3’ | | | | | | | |  |
| *Bgn* | forward: 5’-GTATCCGCAAAGTGCCCAAGG-3’  reverse: 5’-TTTGGGGATGCCAGTGAGCTT-3’ | | | | | | | |  |
| *2610029G23Rik* | forward: 5’-CTCTCTCACCCTCCGGAGTCATA-3’  reverse: 5’-CTCACGGAACTCAGAGTAGATTTGG-3’ | | | | | | | |  |
