# Supplementary material for: The Number of X Chromosomes Causes Sex Differences in Adiposity in Mice
Source: PLoS Genet. 2012 May 10;8(5):e1002709. doi: 10.1371/journal.pgen.1002709 (PMC3349739; doi:10.1371/journal.pgen.1002709)
Supplement: Supplementary file 3 [file pgen.1002709.s001.doc]

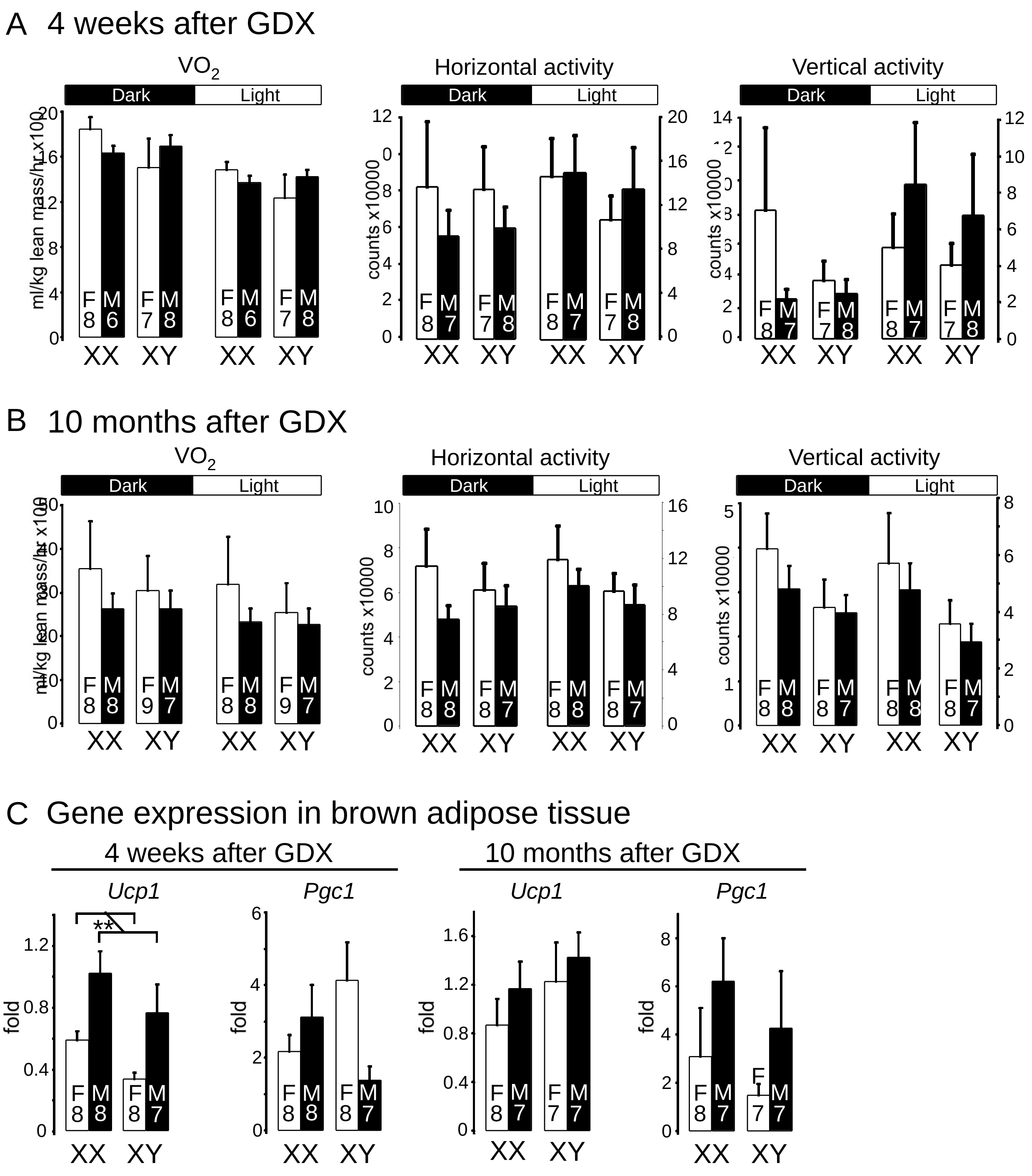

A 4 weeks after GDX
VO2
Horizontal activity
Vertical activity
Dark
Light
ml/kg lean mass/hr x100
counts x10000
XX
XY
B 10 months after GDX
C Gene expression in brown adipose tissue
4 weeks after GDX
10 months after GDX
Ucp1
Pgc1
fold
**

Suppl. Fig. 1
