# Supplementary material for: The Number of X Chromosomes Causes Sex Differences in Adiposity in Mice
Source: PLoS Genet. 2012 May 10;8(5):e1002709. doi: 10.1371/journal.pgen.1002709 (PMC3349739; doi:10.1371/journal.pgen.1002709)
Supplement: Supplementary file 4 [file pgen.1002709.s002.doc]

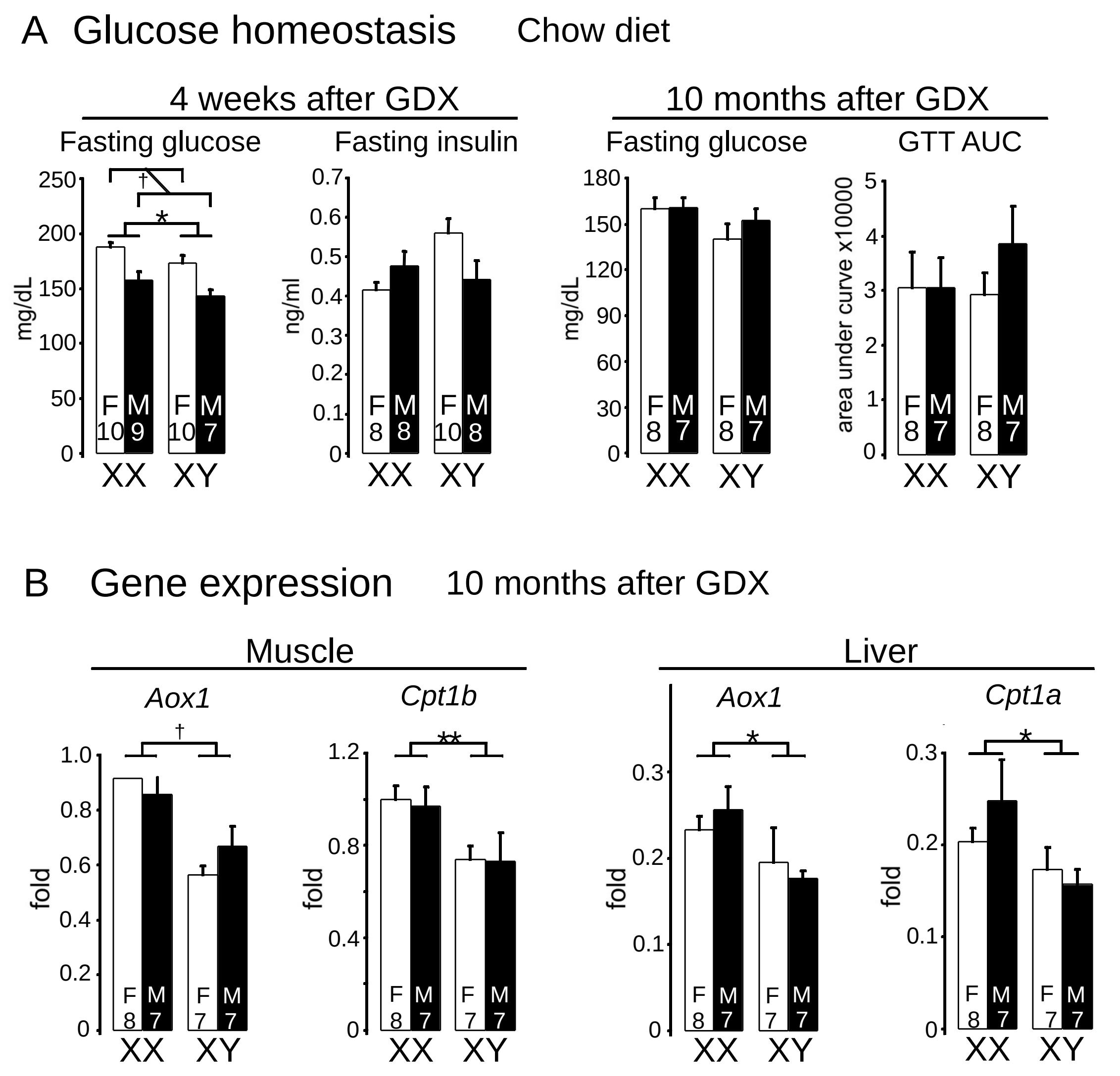
A Glucose homeostasis
Chow diet
4 weeks after GDX
10 months after GDX
Fasting glucose
Fasting insulin
Fasting glucose
GTT AUC
mg/dL
ng/ml
mg/dL
area under curve x10000
XX
XY
F
M
B Gene expression
10 months after GDX
Muscle
Liver
Aox1
Cpt1b
Aox1
Cpt1a
fold

Suppl. Fig. 2
